# Making Science Fun Again: Technology and Heart Failure Therapeutics 2022 Shark Tank

**DOI:** 10.1016/j.jacbts.2022.02.010

**Published:** 2022-04-04

**Authors:** Douglas L. Mann

On behalf of the editors, I am excited to introduce this first special focus edition of *JACC: Basic to Translational Science*: “Technology and Heart Failure Therapeutics 2022 Shark Tank.” Since the inception of *JACC: Basic to Translational Science* in 2016, one of the governing editorial conceits has been to provide an open access format that serves “as a platform for accelerating the translation of new scientific discoveries into new therapies that improve clinical outcomes for patients afflicted with or at risk for cardiovascular disease.”^1^

In collaboration with the Cardiovascular Research Foundation in this special focus issue of *JACC: Basic to Translational Science* we feature 7 presentations by start-up companies who had the opportunity to introduce their concepts to an experienced panel of clinicians, regulatory experts, and investors in the first Technologies for Heart Failure Therapeutics (THT) “Shark Tank” competition. As described in their introductory remarks, Dan Burkhoff, Marty Leon, and Juan Granada mention that the Shark Tank competition was one of the highlights of the inaugural THT meeting held in New York City on February 1-2, 2022.^2^ In this focus issue of *JACC: Basic to Translational Science* each Shark Tank presentation is summarized in a brief research letter that is accompanied by a video link to the presentation by the inventors, followed by a free-wheeling discussion of the presentations by the sharks.∗ The technologies featured in this focus issue range from monitoring devices coupled with novel machine learning algorithms to devices for managing acute decompensated and chronic heart failure.

• AUDICOR Remote Patient Monitoring: FDA Breakthrough Device and Technology for Heart Failure Management (https://www.jacc.org/journal/basic-translational/tht-2022-shark-tank)
• Novel Non-Invasive Biosensors and Artificial Intelligence for Optimized Heart Failure Management (https://www.jacc.org/journal/basic-translational/tht-2022-shark-tank)
• Splanchnic Nerve Ablation for Volume Management in Heart Failure (https://www.jacc.org/journal/basic-translational/tht-2022-shark-tank)
• Synchronized Diaphragmatic Stimulation for the Treatment of Symptomatic Heart Failure: A Novel Implantable Therapy Concept (https://www.jacc.org/journal/basic-translational/tht-2022-shark-tank)
• Novel IVC Doraya Catheter Provides Congestion Relief in Patients With Acute Heart Failure (https://www.jacc.org/journal/basic-translational/tht-2022-shark-tank)
• Cardiac Pulmonary Nerve Stimulation (CPNS): A Novel Treatment for Acute Decompensated Heart Failure (https://www.jacc.org/journal/basic-translational/tht-2022-shark-tank)
• Wireless Power and Water-proof Mechanical Circulatory Assist Device for Ambulatory Heart Failure Patients (https://www.jacc.org/journal/basic-translational/tht-2022-shark-tank)

In addition to the Shark Tank presentations highlighted, in this issue of *JACC: Basic to Translational Science* we also feature a wonderful presentation at THT that was given by Dr Gregg Stone, entitled “Special Challenges of Heart Failure Device Trials” (https://www.jacc.org/journal/basic-translational/tht-2022-shark-tank). His incisive talk illustrates many of the hurdles and challenges faced by inventors and investigators who endeavor to develop new device therapies for heart failure.

In the first Editor’s Page of *JACC: Basic to Translational Science,* we stated that “We will measure our long-term success by the number of new therapies and/or translational avenues first explored in *JACC: Basic to Translational Science.*”^1^ I think that the readership will agree that based on the innovative therapies presented in this focused issue of *JACC: Basic to Translational Science,* we are witnessing the emergence of several new approaches that may eventually lead to improved clinical outcomes for patients afflicted with heart failure. I would be completely remiss if I did not thank Drs Dan Burkhoff, Juan Granada, and Marty Leon, who not only made possible, but also fun (remember that?), to be a part of the THT 2022 Shark Tank. Additionally, I would like to thank the amazing Stephanie Gutch, Senior Director of Digital at Cardiovascular Research Foundation, for all her help in linking the video content from the THT meeting with the *JACC* website, as well as all of the talented staff at *JACC* who made this issue possible, including but in no way limited to Justine Turco (Vice President of Publishing), Monica Payne-Emerson (Executive Managing Editor), and especially to Kim Trevey (Managing Editor) and Jennifer Rapp (Editorial Assistant), who are the 2 very special people who make everything in *JACC: Basic to Translational Science* happen senza errori and on time. As always, I would like to hear your thoughts on this focus issue of *JACC: Basic to Translational Science*, either through social media (*#JACC:BTS*) or by e-mail (JACC@acc.org).
